# Factors Associated with the Uptake of Antenatal Tetanus Toxoids Containing Vaccine by First-Time Mothers in Nigeria: Findings from the 2018 Nigerian Demographic Health Survey

**DOI:** 10.1155/2022/7607993

**Published:** 2022-09-14

**Authors:** Imran Morhason-Bello, Yusuf O. Kareem, Ojone Illah, Joshua O. Akinyemi, Rukiyat Abdus-salam, Olatunji Lawal, Oluwasomidoyin Bello, Gbolahan Obajimi, Isaac F. Adewole

**Affiliations:** ^1^Department of Obstetrics and Gynaecology, Faculty of Clinical Sciences, College of Medicine, University College Hospital, University of Ibadan, Nigeria; ^2^Institute of Advanced Medical Research and Training, College of Medicine, University of Ibadan, Nigeria; ^3^United Nations Population Fund (UNFPA), Abuja, Nigeria; ^4^Department of Women's Health, University College London Hospitals, London, UK; ^5^Department of Epidemiology and Medical Statistics, Faculty of Public Health, University of Ibadan, Nigeria

## Abstract

**Background:**

Maternal and neonatal tetanus remains a public health problem in low-and-middle-income countries despite the increasing investment in tetanus toxoid containing vaccines (TTCV). Nigeria still records fatalities from tetanus, predominantly in women of reproductive age and in newborns. This is largely due to poor access to vaccinations and high rates of unsupervised labour and childbirth. We aim to investigate the antenatal uptake of TTCV and associated factors among first-time pregnant women in Nigeria.

**Methods:**

Data obtained from the 2018 Nigeria Demographic Health Survey (NDHS) was used to generate a list of eligible patients who in the last five years had undergone their first childbirth experience. Data was analysed using univariable and multivariable analyses and reported using a 95% confidence interval.

**Results:**

A total of 3640 participants were included in the analysis. 59.6% (95% CI, 57.6-61.8) of participants had received at least two doses of TTCV. Uptake of TTCV irrespective of current marital status was independently associated with number of and place of antenatal care. Other factors associated with receiving two doses of TTCV in all participants were ownership of mobile phones and region of residence. Among the currently married participants, wealth quintiles, region of residence, and having a polygamous family were additional associated factors.

**Conclusion:**

There was low uptake of the minimal required dosage of TTCV among first-time pregnant women with the lowest uptake in Northern regions relative to Southern regions. We recommend mixed methods studies to further explore the motivation behind TTCV uptake in pregnant women which can help guide future policies and interventions to improve uptake of tetanus immunization in Nigeria.

## 1. Introduction

Maternal and neonatal tetanus remains a public health problem in low-and-middle-income countries, particularly in Southeast Asia and sub-Saharan Africa, with fatality rates sometimes as high as 100% in the newborn [1, 2]. Neonatal tetanus by definition affects newborns within their first month of life whereas maternal tetanus occurs during pregnancy or within six weeks of the end of a pregnancy regardless of outcome—live birth, stillbirth, miscarriage, or abortion [2]. Infection is often a result of unhygienic delivery or pregnancy termination practices, by unskilled attendants [2]. One means of preventing tetanus is via immunization with the tetanus toxoid containing vaccines (TTCV). Maternal and neonatal tetanus can be prevented by vaccinating pregnant women and women of the reproductive age group with TTCV. Immunization with TTCV is a reliable means of preventing tetanus infection, and vaccination of pregnant or reproductive age women will specifically prevent neonatal and maternal tetanus. Thus, antenatal TTCV administration provides a good estimate of the numbers of neonates protected at birth [2, 3].

The true caseload of maternal tetanus worldwide is unknown due to underdiagnosis and underreporting in areas with poor established systems of data collection [2]. As a result, most estimates of maternal tetanus grossly underestimate the true burden of the problem. One way of overcoming this is by using the number of cases of neonatal tetanus to deduce the burden of maternal tetanus [2, 4]. According to the 2018 WHO report, neonatal mortality resulting from tetanus was 25,000 worldwide; this represented an 82% reduction from the 200,000 deaths recorded in 2000. Much of this reduction can be attributed to global and country-level efforts in eliminating neonatal tetanus [4]. The maternal and neonatal tetanus elimination strategy has relied on attaining 80% coverage with two or more doses of TTCV among women of reproductive age, achieving supervision of deliveries by skilled birth attendants in at least 70% of cases, and a robust surveillance programme for new tetanus cases. An increase in the proportion of supervised deliveries by skilled birth attendants from 62% (2000-2005) to 81% (2013-2018) has undoubtedly played a major role in reducing the burden of maternal and neonatal tetanus [2].

In recent years following the 2018 WHO validation, Nigeria has made strides to reduce the burden of maternal and neonatal tetanus by introducing routine immunization with TTCV and increasing the proportion of deliveries that are supervised by skilled attendants [2, 5]. Despite this, Nigeria remains one of thirteen countries yet to achieve elimination of maternal and neonatal tetanus. Maternal immunization with TTCV in Nigeria is largely driven by uptake of antenatal routine immunization; however, many women still miss this opportunity [2, 5]. According to the 2013 National Demographic Health Survey result in Nigeria, antenatal TTCV immunization with at least two doses was highest in Southern regions relative to Northern regions, with the lowest uptake observed in the Northwest [6].

Studies have analysed factors associated with uptake of maternal TTCV during the antenatal period and categorised them into individual, community, and health system factors [7–11]. The individual factors associated with low uptake of TTCV vaccination in women include low socioeconomic class, age at extreme of reproductive period, single marital status, no/low level of education, and no source of income [8, 10]. Poor antenatal clinic (ANC) attendance, distance from antenatal clinic, lack of manpower in clinics, and cost of consumables to administer TTCV are the health system factors that reduce TTCV uptake [10]. Community-related factors include living in rural areas, fixed negative beliefs about vaccinations, and ignorance on vaccination benefits [10, 12]. To achieve the maximal positive impact of TTCV in Nigeria, it is important that data on factors that influence coverage are interrogated regularly for policies and interventions to be developed which address factors mitigating against the uptake of TTCV in women. The goal of this study is to determine antenatal uptake of TTCV and associated factors in among first-time pregnant women in Nigeria using the 2018 Nigeria Demographic and Health Survey (NDHS) dataset and offer recommendations for future policy and investment towards achieving elimination of maternal and neonatal tetanus in the country.

## 2. Materials and Methods

### 2.1. Data Source and Sampling Strategy

The 2018 NDHS dataset is a nationally representative sample of 42,000 households that adopted a stratified two-stage sampling design for recruitment and enrollment. The NDHS classified each locality in Nigerian states and the Federal Capital Territory into urban and rural areas based on population size cut-off points. A locality was classified as urban if there was a population size of 20,000 or more. The first stage sampling—the primary sampling units (PSUs)—included selection of 1400 enumeration areas (EAs) with probability proportional to EA size. The second stage of sampling involved selection of 30 households from every selected cluster using equal probability systematic sampling. The detail of sampling design, implementation, and data collection has been published in the NDHS report [13].

### 2.2. Data Management

#### 2.2.1. Outcomes and Explanatory Variables

The primary outcome of this analysis was the proportion of women that received at least two doses of TTCV. "TTCV uptake" in this analysis refers to the receipt of at least two doses of TTCV. Analysis was restricted to women in the five years preceding the survey with a childbirth experience, with a surviving child. These women were described as “first-time mothers.” Women whose pregnancy resulted in a termination, miscarriage, and stillbirth or whose child died before the survey were excluded as there was no information on the age of these children.

Since maternal and neonatal tetanus elimination has been associated with administration of at least 2 doses of TTCV in pregnancy, eligible women were categorised into two groups as follows: (i) women who received a minimum of 2 doses of TTCV and (ii) women who received no or less than 2 doses of TTCV. Subgroup analysis was conducted based on marital status to further explore the impact of this on TTCV uptake.

Explanatory variables were selected based on review of the literature on maternal and neonatal tetanus elimination policy, program, and interventions. These variables were marital status (never/formerly in union—“not currently in a union” vs. currently married), age of woman at childbirth (<20 years or ≥20 years), number of antenatal visits (no visit, 1 to 3 visits, 4 to 7 visits, and 8 or more visits), place antenatal care was sought (homes/others, government hospitals, government health centres/post or other public facilities, private hospitals, or clinics), wanted pregnancy (No—later or no more, Yes), sex of household head (male or female), household size (3 or less, 4 to 6, and 7 or more), current employment (no, yes), ownership of a mobile phone (no, yes), wealth quintile (poorest, poorer, middle, richer, and richest), highest level of education (no formal education, primary, secondary, and tertiary), exposure to mass media (none at all, at least one of radio, TV, and newspaper), health insurance coverage (no, yes), religion (Christianity, Islam, and others), and geopolitical region (North Central, North East, North West, South East, South South, and South West).

Further explanatory variables were included as part of the subgroup analysis conducted based on marital status. These included type of union (monogamous or polygamous), husband highest level of education (no formal education, primary, secondary, and tertiary), and husband occupation (professional/managerial/technical/skilled, sales or services, agricultural, clerical/skilled or others, and unemployed).

Other variables included were perceived difficulty in accessing healthcare and decision-making power of woman. We used four questions in NDHS on getting medical help to describe perceived difficulty in accessing health care: (1) getting permission to get medical help, (2) getting money needed for treatment, (3) distance to health facility, and (4) not wanting to go to health facilities alone (code = 0, if response is “not a big problem” and code = 1, if “big problem”). Women were categorised into tertiles of low, medium, and high perceived difficulty in accessing health care using principal component analysis (PCA). Decision-making power of women was measured with four questions: (1) who usually decides on respondent's health care? (2) Who usually decides on large household purchases? (3) Who usually decides on visits to family or relatives? (4) Who usually decides what to do with money husband earns? Each of the four questions was coded as follows: “0” if the response was “others or only partner,” “1” if the decision was made “jointly with partner,” and “2” if the decision was made “alone.” A PCA was performed, and collated scores were categorised to the decision-making power of women into tertiles (low, medium, and high).

### 2.3. Data Analysis

We performed a separate analysis on all first-time mothers (irrespective of marital status) and on currently married women. This was to explore the roles of variables that were collected among currently married women and are likely to be associated with maternal health care seeking behaviour. Such variables included decision-making power, polygyny, husband level of education, husband occupation, and age difference between wife and husband.

Descriptive statistics of background characteristics and all analysed variables was performed, in all women who had received at least two doses of TTCV. These were presented as a proportion (percentage) with a 95% confidence interval (CI). Multicollinearity testing was performed by using a variance inflation factor cut-off of five to examine collinearity among variables. There was no evidence of collinearity from variables [14]. Using crude and adjusted ordinal logistic regression, association between background characteristics and adequate TTCV immunization was tested for all first-time mothers and separately for currently married first-time mothers. For currently married first-time mothers only, the following variables were also included in the model: decision-making power, husband level of education, polygyny, husband occupation, and age difference between spouses. In this analysis, we adjusted for the complex survey design (weighting, stratifications, and clustering). All statistical analyses were performed using the STATA program version 16.0 (StataCorp, College Station, Texas, USA) at a 0.05 level of significance.

## 3. Results

### 3.1. Descriptive Characteristics of All First-Time Mothers and Currently Married First-Time Mothers

The weighted percentage summaries of first-time mothers' characteristics (irrespective of current marital status) and their weighted prevalence with 95% CI of taking at least two doses of TTCV are presented in Table 1. The summaries and prevalence with 95% CI of explanatory variables on decision-making power and marital characteristics among currently married women are presented in Table 2. Almost 14% of the mothers were not currently in a union, and 41% were in the adolescent group (<20 years). About one out of five mothers did not attend antenatal services and sought antenatal care from home. In 14% of women, current pregnancy was unwanted, while 45% of women had a household size greater than six. The minority of participants (14%) had a female as the head of household. The proportion of mothers was fairly distributed across the wealth quintiles, with the lowest proportion (17.4%) in the poorest quintile. About 42% of women were unemployed, and almost six in ten had a mobile phone. One in three mothers had no formal education and 44.7% had a secondary education. Half of the mothers experienced low perceived difficulty to access health care, 57.4% resided in urban areas, 33% had no access to mass media, while 2% reported having health insurance coverage. 33.4% of women were from other ethnic minorities; three in ten were Hausas and from the Northwestern region. Over 17% of married mothers were in a polygamous union, and 36.3% had low household decision-making power. Among the married women, 27.5% of husbands had no education and 41.2% had a secondary education. About 3% of their husbands were unemployed, while 31.2% practiced agricultural farming. In the majority of cases, there was a greater than five-year age gap between married women and their husbands.

### 3.2. Uptake of Two or More Doses of TTCV among First-Time Mothers and Currently Married First-Time Mothers

Among first-time mothers, the proportion of women who took at least two doses of TTCV was 59.6% (95% CI: 57.5-61.8) (Table 1). Women currently in a union had a lower uptake (*p* = 59.0%; 95% CI: 56.6-61.3) compared to those not currently in a union (*p* = 63.8%; 95% CI: 58.8-68.5). There was a linear relationship between number of antenatal visits and vaccine uptake, with the least uptake among women who had no antenatal visits (*p* = 3.3%; 95% CI: 2.1-5.1). Adult mothers had a higher TTCV uptake (*p* = 69.8; 95% CI: 67.3-72.3) compared to adolescent mothers (*p* = 45.4; 95% CI: 42.1-48.7).. Higher TTCV uptake was noted in mothers with a currently unwanted pregnancy (*p* = 65.4% 95% CI: 60.7-69.7), those with a household size of four to six (*p* = 63.3%; 95% CI: 59.3-67.1), and families who had a female as the head of household (*p* = 67.2%; 95% CI: 62.4-71.7).

**Figure 1:**
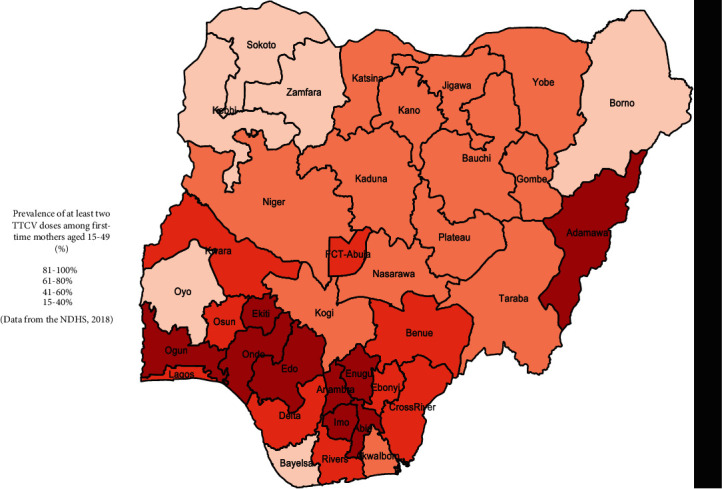
Prevalence of taking at least two doses of TTCV among first-time mothers by states in Nigeria.

Higher TTCV uptake was observed in women who were gainfully employed (*p* = 64.6%; 95% CI: 62.0-67.2), who owned a mobile phone (*p* = 73.1% 95% CI: 70.6-75.4), who had exposure to mass media (*p* = 67.9% 95% CI: 65.5-70.2), and who reported having health insurance coverage (*p* = 76.7% 95% CI: 59.7-88.0). Increasing wealth quintile, increasing maternal household decision-making power, increasing level of both maternal and husband's education, and reducing perceived difficulty in accessing healthcare were linearly associated with increased TTCV uptake. Women in a polygamous union had lower uptake (*p* = 49.7%; 95% CI: 44.4-54.9) compared to those in a monogamous union (*p* = 60.9%; 95% CI: 58.4-63.4). Lower uptake was noted in women residing in rural areas (*p* = 50.8% 95% CI: 48.2-53.5) when compared to those residing in urban areas (*p* = 71.5; 95% CI: 68.0-74.8). Lower uptake was also noted in non-Christian women (*p* = 47.3%; 95% CI: 44.1-50.4). Women in Northern regions had significantly lower TTCV uptake relative to those in Southern regions with the lowest in the Northwest region (*p* = 42.2% and 95% CI: 38.0-46.4). State-to-state variation in TTCV uptake is presented in Figure 1, with the highest uptake in Imo state (94%) and lowest in Sokoto state (17%) (Figure 1).

### 3.3. Factors Associated with Uptake of Two or More Doses of TTCV

The crude and adjusted ordinal logistic regression of factors associated with antenatal TTCV uptake are presented for all first-time mothers (Table 3) and first-time mothers that are currently married (Table 4).

In the adjusted models, factors independently associated with antenatal TTCV uptake in all first-time mothers included antenatal visit attendance, having antenatal care at a health facility, living in the Southeastern region and ownership of a mobile phone. First-time mothers in a polygamous family had higher odds of receiving at least two doses of TTCV (adjusted odds ratio (AOR) = 1.57; 95% CI: 1.06-2.32) relative to those in monogamous families. Within the group of currently married women, there were lesser odds of having two or more doses of TTCV among those from the richer (AOR = 0.95, 95% CI 0.50-0.97) and middle (AOR = 0.67, 95% CI 0.46-0.97) wealth quintiles relative to those from the poorest wealth quintiles.

## 4. Discussion

This study provides information on the uptake of TTCV among first-time mothers in Nigeria including factors that influence the attitude towards the vaccine in this group of women. Our findings showed that the uptake of two or more doses of TTCV fall short of the standard recommended by the WHO for any country that is aspiring to eliminate maternal and neonatal tetanus. We observed significant variation in the prevalence of uptake of two or more doses or more of TTCV among all first-time mothers by marital status, age at childbirth, antenatal clinic attendance, distance to the clinic, desirability of pregnancy, sex of the head of household, family size, occupation, wealth, access to the media, ethnicity, and area of residence. The prevalence of TTCV uptake among currently married first-time mothers was additionally associated with the ability of women to make decisions in the household, age difference between spouses, and husband's level of education.

The low uptake of at least two doses of TTCV among first-time mothers is worrisome. The implication of course being that a large proportion of neonates delivered by inadequately immunized mothers are not protected against tetanus infection. Generally, compared to women who have had previous pregnancies, first-time pregnant women are given health talks, and in some clinics, they are offered extra antenatal counselling sessions. Previous studies have reported on individual and community-level factors associated with uptake of TTCV in Nigeria among women with different categories of pregnancy and childbirth [11, 15, 16]. Although the population investigated in this study was limited to first-time mothers, we observed that some factors associated with the uptake of two doses of TTCV shared similarities with studies conducted among other categories of women in Nigeria [11, 15]. Two factors consistently associated with higher odds of having at least two doses of TTCV are a high number of antenatal visits and location in the Southern region of Nigeria. In line with this, this analysis, showed that higher antenatal clinic attendance, antenatal care at a health facility, and Southern regional location were all associated with higher odds of receiving at least two doses of TTCV in all first-time mothers and in first-time mothers that are currently married.

Surprisingly, regression analysis showed that women in polygamous unions had higher odds of TTCV uptake compared to women in monogamous unions in currently married first-time mothers. It is plausible that in-house discussions and/or counselling and motivation from other women within the family who have antenatal and childbirth experience contribute to this. Another interesting finding was the lower uptake of TTCV in first-time mothers in the middle and richer wealth quintiles compared to those in the poorest wealth quintiles. TTCV is predominantly administered at the public primary healthcare/infant welfare clinics in Nigeria, mostly patronized by people in the low socioeconomic class [17]; this might explain the neglect of TTCV in women of higher socioeconomic class. Nonetheless, this finding will require further exploration in future studies employing mixed methods to determine whether family wealth has a causal or artefactual association with antenatal TTCV uptake and understand the underlying reasons. Finally, having a large household size of seven and above was associated with lower odds of receiving at least two doses of TTCV. Large family size has been associated with a delay in accessing childhood immunization. Some authors have posited that the domestic and financial pressure of a large family size distracts women from accessing healthcare for themselves, their children, and family members [18].

One limitation of this study is that secondary data was used to model risk factors associated with uptake of TTCV in the study population. It is therefore possible that we may have omitted other important explanatory variables that influence TTCV uptake. As this study is cross-sectional in design, it is impossible to draw causality association between risk factors and outcome measures. Despite these limitations, this study has provided unique information on the profile of first-time pregnant women that are likely to receive two doses of TTCV during their antenatal period. The survey data was collected from a nationally representative sample of women. Based on these findings, early identification of women that are unlikely to receive optimal TTCV in pregnancy could help health workers and program planners to device counselling techniques and interventions to encourage TTCV uptake in these women.

In conclusion, this analysis has demonstrated that there are several variables that are associated with low uptake of TTCV among first-time mothers, including huge regional variation, with the lowest uptake in the Northwestern region. It is important to design culturally sensitive messages that will motivate these groups of women on the importance of TTCV in reducing the mortality and morbidity of tetanus in them and their unborn babies. We recommend future studies employ a mixed methods design to explore reasons behind low uptake of TTCV, particularly in first-time mothers who are either in the early or middle period of their reproductive career.

## Figures and Tables

**Table 1:** Descriptive characteristics of first-time mothers who took at least two doses of TTCV, NDHS 2018.

Variable	All first-time mothers *N* = 3640	Currently married mothers *N* = 3058	All first-time mothers ≥2 tetanus	Currently married mothers ≥2 tetanus
	Frequency (%)	Frequency (%)	Prevalence (%) (95% CI)	Prevalence (%) (95% CI)
			59.6 (57.5-61.8)	59.0 (56.6-61.3)
*Marital status*			*p* = 0.079	
Formerly/never in union	501 (13.8)	NA	63.8 (58.8-68.5)	NA
Currently in union	3139 (86.2)	3058 (100)	59.0 (56.6-61.3)	59.0 (56.6-61.3)
*Age at childbirth (years)*			*p* < 0.001	*p* < 0.001
<20 (adolescent)	1518 (41.7)	1274 (41.7)	45.4 (42.1-48.7)	43.9 (40.3-47.5)
≥20 (adult)	2122 (58.3)	1784 (58.3)	69.8 (67.3-72.3)	69.7 (66.9-72.4)
*Antenatal visits*			*p* < 0.001	*p* < 0.001
No visit	679 (18.7)	574 (18.8)	3.3 (2.1-5.1)	2.6 (1.5-4.4)
1-3 visits	577 (15.8)	487 (15.9)	50.3 (45.3-55.3)	49.2 (43.8-54.6)
4-7 visits	1440 (39.5)	1208 (39.5)	74.6 (71.6-77.4)	73.8 (70.4-77.0)
≥8 visits	945 (26.0)	790 (25.8)	83.0 (79.7-85.9)	
*Place ANC sought*			*p* < 0.001	*p* < 0.001
Home or others	805 (22.1)	675 (22.1)	11.5 (8.8-14.8)	10.2 (7.6-13.7)
Government hospitals	913 (25.1)	780 (25.5)	75.3 (71.3-78.8)	75.6 (71.4-79.3)
Government health centres or post	1282 (35.2)	1062 (34.7)	71.1 (68.1-73.9)	69.5 (66.3-72.5)
Private hospitals/clinics	640917.6)	540 (17.7)	74.8 (70.0-79.1)	75.2 (69.9-79.9)
*Wanted pregnancy*			*p* = 0.012	*p* < 0.001
No	508 (14.0)	217 (7.1)	65.4 (60.7-69.7)	71.6 (64.7-77.7)
Yes	3132 (86.0)	2841 (92.9)	58.7 (56.3-61.0)	58.0 (55.5-60.4)
*Sex of household head*			*p* = 0.001	*p* = 0.003
Male	3130 (86.0)	2784 (91.0)	58.4 (56.0-60.7)	57.9 (55.5-60.4)
Female	510 (14.0)	274 (9.0)	67.2 (62.4-71.7)	69.4 (62.5-75.4)
*Household size*			*p* < 0.001	*p* = 0.002
≤3	2004 (55.1)	1823 (59.6)	60.7 (57.9-63.4)	60.0 (57.1-62.8)
4-6	872 (24.0)	671 (21.9)	63.3 (59.3-67.1)	62.6 (58.1-66.9)
≥7	764 (21.0)	564 (18.4)	52.7 (48.3-57.0)	51.2 (46.2-56.3)
*Currently working*			*p* < 0.001	*p* < 0.001
No	1527 (42.0)	1327 (43.4)	52.7 (49.2-56.1)	51.6 (47.9-55.4)
Yes	2113 (58.0)	1731 (56.6)	64.6 (62.0-67.2)	64.6 (61.7-67.4)
*Own a mobile phone*			*p* < 0.001	
No	1523 (41.8)	1336 (43.7)	40.9 (37.8-44.1)	39.9 (36.6-43.3)
Yes	2117 (58.2)	1722 (56.3)	73.1 (70.6-75.4)	73.8 (71.1-76.3)
*Wealth quintiles*			*p* < 0.001	*p* < 0.001
Poorest	633 (17.4)	549 (17.9)	35.6 (31.3-40.2)	34.5 (30.1-39.3)
Poorer	770 (21.1)	662 (21.7)	49.5 (45.2-53.9)	47.6 (42.9-52.2)
Middle	711 (19.5)	566 (18.5)	57.8 (53.7-61.9)	55.8 (51.3-60.1)
Richer	754 (20.7)	616 (20.2)	71.0 (66.8-74.9)	72.9 (68.3-77.0)
Richest	772 (21.2)	664 (21.7)	80.0 (75.8-83.6)	80.3 (75.7-84.2)
*Highest level of education*			*p* < 0.001	*p* < 0.001
None	1176 (32.3)	1091 (35.7)	36.2 (32.6-39.9)	36.0 (32.4-39.7)
Primary	371 (10.2)	294 (9.6)	59.1 (51.9-66.0)	58.5 (50.1-66.3)
Secondary	1628 (44.7)	1267 (41.4)	69.6 (66.7-72.4)	70.7 (67.5-73.7)
Higher	465 (12.8)	405 (13.3)	84.3 (79.1-88.4)	84.5 (78.8-88.9)
*Exposure to mass media*			*p* < 0.001	*p* < 0.001
Not at all	1203 (33.0)	1046 (34.2	42.9 (39.4-46.4)	40.9 (37.3-44.7)
Have access to at least one	2437 (77.0)	2012 (65.8)	67.9 (65.5-70.2)	68.3 (65.8-70.8)
*Covered by health insurance*			*p* = 0.041	*p* = 0.008
No	3572 (98.1)	3000 (98.1)	59.3 (57.1-61.5)	58.6 (56.2-60.9)
Yes	68 (1.9)	58 (1.9)	76.7 (59.7-88.0)	80.1 (64.3-90.0)
*Perceived difficulty to access health care*			*p* < 0.001	*p* < 0.001
Low	1803 (49.5)	1548 (50.6)	65.8 (62.7-68.8)	65.6 (62.3-68.8)
Middle	791 (21.7)	583 (19.1)	62.3 (58.0-66.4)	61.9 (57.1-66.4)
High	1046 (28.7)	927 (30.3)	47.0 (43.0-51.1)	46.0 (41.8-50.3)
*Residence*			*p* < 0.001	*p* < 0.001
Urban	1552 (42.6)	1273 (41.6)	71.5 (68.0-74.8)	71.8 (67.8-75.4)
Rural	2088 (57.4)	1785 (58.4)	50.8 (48.2-53.5)	49.8 (47.0-52.6)
*Religion*			*p* < 0.001	*p* < 0.001
Christians	1741 (47.8)	1318 (43.1)	73.1 (70.4-75.7)	75.0 (72.0-77.8)
Islam and others	1899 (52.2)	1740 (56.9)	47.3 (44.1-50.4)	46.8 (43.5-50.1)
*Ethnicity*			*p* < 0.001	p < 0.001
Fulani	242 (6.7)	229 (7.5)	31.7 (25.1-39.3)	31.1 (24.5-38.5)
Hausa	1082 (29.7)	1002 (32.8)	44.7 (40.6-49.0)	44.5 (40.3-48.7)
Igbo	548 (15.0)	416 (13.6)	83.9 (79.7-87.4)	85.0 (80.0-88.9)
Yoruba	552 (15.2)	460 (15.0)	71.4 (64.6-77.4)	70.5 (62.9-77.1)
Other ethnic minorities	1216 (33.4)	951 (31.1)	62.1 (58.7-65.5)	64.0 (60.2-67.6)
*Region*			*p* < 0.001	*p* < 0.001
North Central	555 (15.2)	472 (15.4)	56.9 (51.7-61.9)	57.8 (52.5-63.0)
North East	571 (15.7)	476 (15.6)	53.3 (47.8-58.8)	51.2 (45.4-56.9)
North West	1033 (28.4)	963 (31.5)	42.2 (38.0-46.4)	42.1 (37.9-46.4)
South East	424 (11.7)	319 (10.4)	85.7 (81.8-88.8)	87.7 (83.6-90.9)
South South	404 (11.1)	285 (9.3)	70.7 (65.7-75.3)	75.9 (70.5-80.5)
South West	653 (17.9)	544 (17.8)	71.3 (65.7-76.2)	70.9 (64.7-76.4)

NA: not applicable.

**Table 2:** Marriage characteristics of currently married first-time mothers who took at least two doses of TTCV, NDHS 2018.

Variable	*N* = 3058	≥2 tetanus
	Frequency (%)	Prevalence (%) (95% CI)
*Decision-making power*		*p* < 0.001
Low	1107 (36.3)	47.8 (44.0-51.7)
Middle	1062 (34.9)	61.1 (57.5-64.7)
High	879 (28.8)	70.2 (65.7-74.3)
*Husband highest level of education*		*p* < 0.001
None	830 (27.5)	31.0 (27.1-35.3)
Primary	314 (10.4)	54.7 (47.5-61.8)
Secondary	1242 (41.2)	69.2 (65.7-72.5)
Tertiary	631 (20.9)	78.4 (73.8-82.4)
*Polygamous*		*p* < 0.001
No	2528 (82.8)	60.9 (58.4-63.4)
Yes	525 (17.2)	49.7 (44.4-54.9)
*Difference in age between partner*		*p* = 0.004
Wife older or same age	47 (1.5)	63.2 (46.1-77.6)
Husband 1-5 years older	873 (28.6)	65.0 (60.3-69.4)
Husband 6-10 years older	1154 (37.7)	58.0 (54.3-61.2)
Husband >10 years older	984 (32.2)	54.8 (50.8-58.6)
*Husband occupation*		*p* < 0.001
Professional or skilled	503 (16.5)	74.0 (68.8-78.7)
Sales or services	882 (28.9)	60.9 (56.5-65.1)
Agricultural	951 (31.2)	43.7 (39.9-47.6)
Clerical	77 (2.53)	74.7 (61.1-84.8)
Unskilled and others	544 (17.8)	67.6 (62.5-72.2)
Not working	96 (3.1)	54.5 (41.9-66.5)

**Table 3:** Crude and adjusted ordinal logistic regression of first-time mothers who took at least two doses of TTCV.

Variable	All first-time mothers	
	OR (95% CI)	AOR (95% CI)
*Marital status*		
Formerly/never in union	1.0 Reference	1.0 Reference
Currently in union	0.81 (0.65-1.02)^∗^	1.08 (0.77-1.51)
*Age at child birth (years)*		
<20 (adolescent)	1.0 Reference	1.0 Reference
≥20 (adult)	2.78 (2.33-3.33)^∗∗∗^	1.19 (0.91-1.49)
*Antenatal visits*		
No visit	1.0 Reference	1.0 Reference
1-3 visits	29.83 (18.05-49.27)^∗∗∗^	8.69 (4.23-17.88)^∗∗∗^
4-7 visits	86.58 (53.00-141.41)^∗∗∗^	23.29 (11.30-47.99)^∗∗∗^
≥8 visits	144.09 (86.95-238.77)^∗∗∗^	35.15 (16.79-73.56)^∗∗∗^
*Place ANC sought*		
Home or others	1.0 Reference	1.0 Reference
Government hospitals	23.48 (16.45-33.51)^∗∗∗^	3.67 (2.04-6.62)^∗∗∗^
Government health centres or post	18.98 (13.69-26.30)^∗∗∗^	3.43 (1.98-5.92)^∗∗∗^
Private hospitals/clinics	22.94 (15.63-33.66)^∗∗∗^	1.81 (1.01-3.21)^∗∗^
*Wanted pregnancy*		
No	1.0 Reference	1.0 Reference
Yes	0.75 (0.60-0.94)^∗∗^	0.92 (0.66-1.30)
*Sex of household head*		
Male	1.0 Reference	1.0 Reference
Female	1.46 (1.16-1.84)^∗∗^	1.05 (0.76-1.44)
*Household size*		
≤3	1.0 Reference	1.0 Reference
4-6	1.12 (0.92-1.35)	0.98 (0.77-1.26)
≥7	0.72 (0.58-0.89)^∗∗^	0.87 (0.66-1.14)
*Currently working*		
No	1.0 Reference	1.0 Reference
Yes	1.64 (1.37-1.97)^∗∗∗^	0.95 (0.76-1.20)
*Own a mobile phone*		
No	1.0 Reference	1.0 Reference
Yes	3.93 (3.23-4.67)^∗∗∗^	1.61 (1.26-2.06)^∗∗∗^
*Wealth quintiles*		
Poorest	1.0 Reference	1.0 Reference
Poorer	1.78 (1.38-2.29)^∗∗∗^	1.15 (0.85-1.54)
Middle	2.48 (1.92-3.21)^∗∗∗^	0.88 (0.63-1.24)
Richer	4.43 (3.35-5.86)^∗∗∗^	1.24 (0.83-1.86)
Richest	7.23 (5.30-9.87)^∗∗∗^	1.21 (0.77-1.91)
*Highest level of education*		
None	1.0 Reference	1.0 Reference
Primary	2.55 (1.83-3.57)^∗∗∗^	1.13 (0.77-1.66)
Secondary	4.05 (3.28-4.99)^∗∗∗^	1.04 (0.71-1.52)
Higher	9.50 (6.48-13.93)^∗∗∗^	1.38 (0.81-2.37)
*Exposure to mass media*		
Not at all	1.0 Reference	1.0 Reference
Have access to at least one type of media	2.82 (2.37-3.36)^∗∗∗^	0.84 (0.65-1.09)
*Covered by health insurance*		
No	1.0 Reference	1.0 Reference
Yes	2.26 (1.01-5.04)^∗∗^	1.54 (0.70-3.36)
*Perceived difficulty to access healthcare*		
Low	1.0 Reference	1.0 Reference
Middle	0.85 (0.68-1.06)	1.11 (0.84-1.45)
High	0.48 (0.39-0.59)^∗∗∗^	0.80 (0.61-1.05)
*Residence*		
Urban	1.0 Reference	1.0 Reference
Rural	0.42 (0.34-0.50)	1.21 (0.90-1.62)
*Religion*		
Christians	1.0 Reference	1.0 Reference
Islam and others	0.33 (0.27-0.40)^∗∗∗^	0.87 (0.59-1.28)
*Ethnicity*		
Fulani	1.0 Reference	1.0 Reference
Hausa	1.74 (1.20-2.52)^∗∗^	1.44 (0.88-2.36)
Igbo	11.22 (7.28-17.30)^∗∗∗^	1.43 (0.64-3.19)
Yoruba	5.37 (3.41-8.46)^∗∗∗^	1.27 (0.65-2.49)
Other ethnic minorities	3.53 (2.46-5.06)^∗∗∗^	1.42 (0.87-2.32)
*Region*		
North Central	1.0 Reference	1.0 Reference
North East	0.87 (0.64-1.18)	1.09 (0.73-1.62)
North West	0.55 (0.42-0.72)^∗∗∗^	0.61 (0.39-0.97)^∗∗^
South East	4.54 (3.19-6.46)^∗∗∗^	2.47 (1.18-5.15)^∗∗^
South South	1.83 (1.34-2.50)^∗∗∗^	1.42 (0.87-2.32)
South West	1.88 (1.35-2.62)^∗∗∗^	0.73 (0.43-1.24)

^∗∗∗^ *p* < 0.001; ^∗∗^*p* < 0.05; ^∗^*p* < 0.10.

**Table 4:** Crude and adjusted ordinal logistic regression of currently married first-time mothers who took at least two doses of TTCV.

Variable	Currently married first time mothers	
	OR (95% CI)	AOR (95% CI)
*Age at child birth (years)*		
<20 (adolescent)	1.0 Reference	1.0 Reference
≥20 (adult)	2.95 (2.42-3.60)^∗∗∗^	1.10 (0.83-1.45)
*Antenatal visits*		
No visit	1.0 Reference	1.0 Reference
1-3 visits	36.60 (20.17-66.40)^∗∗∗^	9.89 (4.19-23.35)^∗∗∗^
4-7 visits	106.73 (59.16-192.53)^∗∗∗^	25.0 (10.42-59.96)^∗∗∗^
≥8 visits	187.57 (102.90-341.91)^∗∗∗^	42.68 (17.27-105.49)^∗∗∗^
*Place ANC sought*		
Home or others	1.0 Reference	1.0 Reference
Government hospitals	27.18 (18.29-40.38)^∗∗∗^	4.06 (2.10-7.85)^∗∗∗^
Government health centres or post	20.02 (13.90-28.85)^∗∗∗^	3.78 (2.03-7.03)^∗∗∗^
Private hospitals/clinics	26.71 (17.49-40.77)^∗∗∗^	2.11 (1.11-4.03)^∗∗^
*Wanted pregnancy*		
No	1.0 Reference	1.0 Reference
Yes	0.55 (0.39-0.76)^∗∗∗^	0.76 (0.48-7.85)
*Sex of household head*		
Male	1.0 Reference	1.0 Reference
Female	1.64 (1.19-2.27)^∗∗^	1.17 (0.76-1.80)
*Household size*		
≤3	1.0 Reference	1.0 Reference
4-6	1.11 (0.91-1.37)	0.87 (0.65-1.17)
≥7	0.70 (0.55-0.89)^∗∗^	0.69 (0.49-0.96)^∗∗^
*Currently working*		
No	1.0 Reference	1.0 Reference
Yes	1.71 (1.40-2.08)^∗∗∗^	0.98 (0.75-1.28)
*Own a mobile phone*		
No	1.0 Reference	1.0 Reference
Yes	4.23 (3.50-5.12)^∗∗∗^	1.78 (1.35-2.33)
*Wealth quintile*		
Poorest	1.0 Reference	1.0 Reference
Poorer	1.72 (1.31-2.25)^∗∗∗^	0.92 (0.67-1.28)
Middle	2.39 (1.82-3.13)^∗∗∗^	0.67 (0.46-0.97)^∗∗^
Richer	5.09 (3.76-6.89)^∗∗∗^	0.95 (0.50-0.97)
Richest	7.74 (5.52-10.83)^∗∗∗^	0.87 (0.51-1.49)
*Highest level of education*		
None	1.0 Reference	1.0 Reference
Primary	2.50 (1.73-3.63)^∗∗∗^	0.93 (0.60-1.25)
Secondary	4.29 (3.43-5.37)^∗∗∗^	0.77 (0.48-1.22)
Higher	9.71 (6.43-14.67)^∗∗∗^	1.03 (0.53-2.01)
*Exposure to mass media*		
Not at all	1.0 Reference	1.0 Reference
Have access to at least one type of media	3.11 (2.58-3.76)^∗∗∗^	0.93 (0.70-1.25)
*Covered by health insurance*		
No	1.0 Reference	1.0 Reference
Yes	2.85 (1.27-6.38)^∗∗^	1.95 (0.72-5.33)
*Perceived difficulty to access healthcare*		
Low	1.0 Reference	1.0 Reference
Middle	0.85 (0.67-1.08)	1.12 (0.83-1.52)
High	0.44 (0.35-0.56)^∗∗∗^	0.74 (0.55-1.00)^∗∗^
*Residence*		
Urban	1.0 Reference	1.0 Reference
Rural	0.39 (0.31-0.49)^∗∗∗^	1.24 (0.90-1.72)
*Religion*		
Christians	1.0 Reference	1.0 Reference
Islam and others	0.29 (0.24-0.36)^∗∗∗^	0.95 (0.62-1.45)
*Ethnicity*		
Fulani	1.0 Reference	1.0 Reference
Hausa	1.78 (1.23-2.57)^∗∗^	1.54 (0.92-2.58)
Igbo	12.56 (7.80-20.23)^∗∗∗^	1.46 (0.61-3.51)
Yoruba	5.31 (3.30-8.53)^∗∗∗^	1.19 (0.58-2.42)
Other ethnic minorities	3.95 (2.73-5.70)^∗∗∗^	1.47 (0.87-2.48)
*Region*		
North Central	1.0 Reference	1.0 Reference
North East	0.76 (0.56-1.05)^∗^	0.90 (0.58-1.40)
North West	0.53 (0.40-0.70)^∗∗∗^	0.51 (0.31-0.84)^∗∗^
South East	5.20 (3.50-7.75)^∗∗∗^	3.22 (1.42-7.33)^∗∗^
South South	2.29 (1.61-3.25)^∗∗∗^	1.65 (0.90-3.02)
South West	1.78 (1.24-2.54)^∗∗^	0.68 (0.38-1.22)
*Household decision-making*		
Low	1.0 Reference	1.0 Reference
Middle	1.72 (1.39-2.12)^∗∗∗^	1.03 (0.78-1.36)
High	2.57 (1.96-3.38)^∗∗∗^	0.91 (0.65-1.29)
*Husband highest level of education*		
None	1.0 Reference	1.0 Reference
Primary	2.68 (1.88-3.84)^∗∗∗^	1.19 (0.76-1.86)
Secondary	5.00 (3.88-6.44)^∗∗∗^	1.49 (0.99-2.25)^∗^
Tertiary	8.07 (5.85-11.15)^∗∗∗^	1.77 (1.05-2.97)^∗∗^
*Polygamous*		
No	1.0 Reference	1.0 Reference
Yes	0.63 (0.50-0.80)^∗∗∗^	1.57 (1.06-2.33)^∗∗^
*Difference in age between partner*		
Wife older or same age	1.42 (0.70-2.88)	0.57 (0.26-1.22)
Husband 1-5 years older	1.54 (1.19-1.99)^∗∗^	1.03 (0.74-1.45)
Husband 6-10 years older	1.13 (0.93-1.37)	1.08 (0.80-1.45)
Husband > 10 years older	1.0 Reference	1.0 Reference
*Husband occupation*		
Professional or Skilled	2.38 (1.33-4.25)^∗∗^	0.62 (0.31-1.25)
Sales or services	1.30 (0.77-2.21)	0.65 (0.33-1.27)
Agricultural	0.65 (0.38-1.10)	0.75 (0.39-1.46)
Clerical/unskilled and others	1.81 (1.05-3.14)^∗∗^	0.76 (0.38-1.54)
Not working	1.0 Reference	1.0 Reference

^∗∗∗^ *p* < 0.001; ^∗∗^*p* < 0.05; ^∗^*p* < 0.10.

## Data Availability

Interested parties can obtain data from the Demographic and Health Surveys (DHS) website.
